# Green Leaf Volatiles: A Plant’s Multifunctional Weapon against Herbivores and Pathogens

**DOI:** 10.3390/ijms140917781

**Published:** 2013-08-30

**Authors:** Alessandra Scala, Silke Allmann, Rossana Mirabella, Michel A. Haring, Robert C. Schuurink

**Affiliations:** Department of Plant Physiology, Swammerdam Institute for Life Sciences, Science Park 904, Amsterdam 1098 XH, The Netherlands; E-Mails: a.scala@uva.nl (A.S.); S.Allmann@uva.nl (S.A.); rossanamirabella@gmail.com (R.M.); M.A.Haring@uva.nl (M.A.H.)

**Keywords:** secondary metabolites, green leaf volatiles, phytohormones, pathogen, plant-to-plant communication, priming, indirect defenses

## Abstract

Plants cannot avoid being attacked by an almost infinite number of microorganisms and insects. Consequently, they arm themselves with molecular weapons against their attackers. Plant defense responses are the result of a complex signaling network, in which the hormones jasmonic acid (JA), salicylic acid (SA) and ethylene (ET) are the usual suspects under the magnifying glass when researchers investigate host-pest interactions. However, Green Leaf Volatiles (GLVs), C_6_ molecules, which are very quickly produced and/or emitted upon herbivory or pathogen infection by almost every green plant, also play an important role in plant defenses. GLVs are semiochemicals used by insects to find their food or their conspecifics. They have also been reported to be fundamental in indirect defenses and to have a direct effect on pests, but these are not the only roles of GLVs. These volatiles, being probably one of the fastest weapons exploited, are also able to directly elicit or prime plant defense responses. Moreover, GLVs, via crosstalk with phytohormones, mostly JA, can influence the outcome of the plant’s defense response against pathogens. For all these reasons GLVs should be considered as co-protagonists in the play between plants and their attackers.

## 1. Introduction

Plants are sessile organisms that are continuously subjected to a large number of different stresses. During some of these challenging conditions, GLVs are produced and/or emitted. GLVs consist of a family of C_6_ compounds, including aldehydes, alcohols and esters, and this evocative name originates from their fragrance which is perceived by humans similarly to the one of grass clippings [1,2].

These leafy volatiles originate in the hydroperoxide lyase (HPL) branch of the oxylipin pathway and they are formed from fatty acids [1]. GLVs are almost ubiquitously made by green plants [2] and their increased release can be caused by abiotic stimuli [2–5], by herbivores [6,7] or pathogens [8–10]. Their emission is further influenced by environmental conditions such as soil humidity, fertilization and temperature [11]. While un-stressed plants emit only traces of GLVs [7,12] they can be rapidly formed, within a few seconds upon stress [13]. GLV emission is transient but can be maintained for days upon herbivory or repetitive wounding [14]. The rapidity of production and ability to be boosted continuously are good features of information-carrying molecules and in fact, many studies underline that GLVs have this feature.

Indeed, so far, GLVs have been reported to be messengers in tritrophic interactions and in “plant communication”. The first one is also described as “plant indirect defenses”, by which the plant tries to dispose of herbivores by pointing out their presence to their predators. The second one has to be considered not only as a set of information that the emitter releases to neighboring plants, but also to distal parts of the emitter [10,15–18].

GLVs are also involved in inducing plant defenses and in triggering “priming”, a state that prepares the plant to respond in an accelerated and/or augmented way to herbivory or pathogen attack [19–21].

Another aspect that illustrates the versatility and the importance of these volatile compounds is that GLVs, by effecting mainly JA signaling or levels, can change the phytohormone dynamic equilibrium [22–26]. This crosstalk between phytohormones is essential in the regulation and organization of plant responses to biotic and abiotic stresses that often occur simultaneously.

Since GLVs are involved in many aspects of plant adaptation to their environment, they can be defined as multifaceted molecules that are produced upon various perturbations of the plant system and help plants to survive in a hostile environment. In this review, we want to outline the physiological importance of GLVs and to point out that they play key roles in plant defense responses.

## 2. Green Leaf Volatile Biosynthesis

**Figure 1:**
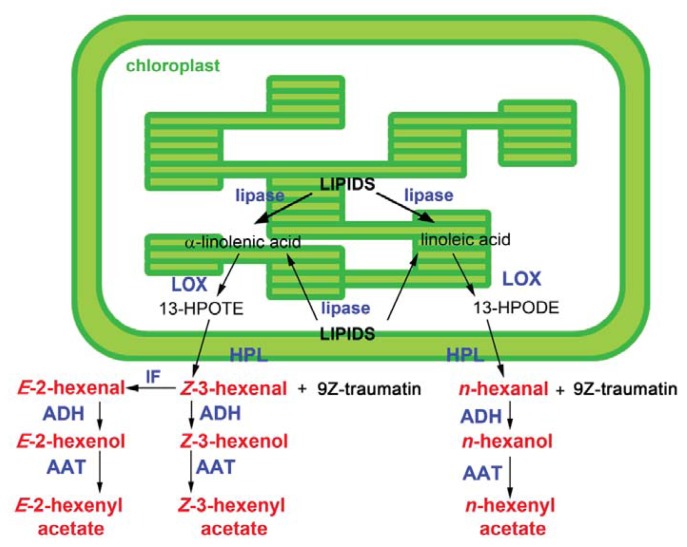
Green Leaf Volatile (GLV) biosynthesis. Lipase(s) release(s) α-linolenic and linoleic acid from galactolipids. 13-lipoxygenases (LOXs) catalyze the addition of oxygen to α-linolenic acid to form 13(S)-hydroperoxy 9*Z*,11*E*,15*Z*-octadecatrienoic acid (13-HPOTE), in Section 2 referred to as 13-hydroperoxide. 13-HPOTE is converted to *Z*-3-hexenal and 9*Z*-traumatin by 13-HPL (HPL). An isomerization factor, (3*Z*):(2*Z*)-enal isomerase (IF) is responsible for converting *Z*-3-hexenal into its isomer, *E*-2-hexenal. Furthermore, this reaction occurs spontaneously. *Z*-3-hexenal and *E*-2-hexenal are reduced to *Z*-3-hexenol and *E*-2-hexenol, respectively, by alcohol dehydrogenase(s) (ADH). *Z*-3-hexenol and *E*-2-hexenyl acetate are converted to *Z*-3-hexenyl acetate and *E*-2-hexenyl acetate by alcohol acyltransferase(s) (AAT). 13-lipoxygenases (LOXs) catalyze the addition of oxygen to linoleic acid to form 13(S)-hydroperoxy 9*Z*,11*E*-octadecadienoic (13-HPODE), in section 2 referred to as 13-hydroperoxide. 13-HPODE is converted to *n*-hexanal and 9*Z*-traumatin by 13-HPL. *n*-hexanal is converted by alcohol dehydrogenase(s) to *n*-hexanol, which is converted to *n*-hexenyl acetate by alcohol acyl transferase(s).

GLVs are synthesized via the hydroperoxide lyase (HPL) branch of the oxylipin pathway. Oxylipins are oxygenated products of fatty acids and regulate many defense and developmental pathways in plants [27]. The first step of this metabolic route is catalyzed by lipases that deacylate galactolipids to release free α-linolenic [28], from which JA is also derived [29] or linoleic acid (Figure 1). However, which specific lipase(s) are involved in the activation of the GLV biosynthesis is still unknown. So far the lipases characterized in *Arabidopsis thaliana* and *Nicotiana attenuata* are mainly involved into JA biosynthesis. In Arabidopsis phospholipase A (PLA) participates in JA formation while DEFECTIVE IN ANTHER DEHISCENCE 1 (DAD1) acts only in the late wound JA accumulation [30]. *N. attenuata* GLA1, a lipase located in plastids, is a major supplier of fatty acids for JA biosynthesis after wounding and herbivory, but not for GLV biosynthesis [31]. In rice, the silencing of two phospolipases D (PLDs), *PLDα4* and *PLDα5*, decreases the expression of allene oxide cyclase (*AOC*) and *HPL3*, involved in the biosynthesis of JA and GLVs, respectively [32]. These two lipases are involved in the activation of both JA and GLV biosynthesis, but there might be other lipases involved, since levels of JA and GLVs are not completely repressed in rice plants with reduced *PLDα4* and *PLDα5* expression (as-pld).

In the second enzymatic step, α-linolenic and linoleic acids, released by the lipases, become substrates to non-heme, iron-binding enzymes, C13-lipoxygenases (13-LOX, here further referred to as LOX), which catalyze the addition of oxygen to form, respectively, linolenic acid 13-hydroperoxide, also an intermediate in the JA biosynthetic pathway [29] and linoleic acid 13-hydroperoxide (13-hydroperoxides).

While only few studies exist that show pathway specificity for lipases much more data is available describing such specificity for lipoxygenases. In potato, two different wound inducible lipoxygenases, LOX-H1 and LOX-H3, supply hydroperoxides to the GLV and JA pathway branches respectively. Reduced *LOX-H1* expression shows a clear reduction of GLVs while the levels of these volatiles are unaffected in as-lox-h3 plants [33]. In tomato, the silencing of *LOXC* leads to a significant reduction of GLVs in leaves and fruits while the silencing of two other lipoxygenases, *LOXA* and *LOXB*, does not show any effect on GLV levels [34]. In *N. attenuata* two different LOXs have been identified, of which NaLOX2 specifically supplies hydroperoxides for GLV biosynthesis and NaLOX3 for JA biosynthesis [35]. In a recent paper, two LOXs of maize, ZmLOX10 and ZmLOX8, have been shown to be specialized in providing substrate for the GLV and the JA biosynthesis pathways, respectively [22]. However, there is also one example of a rice chloroplast localized LOX whose silencing affects both GLV and JA levels [36]. LOXs are reported to be chloroplast localized in several species [33,37–40].

For completeness, it must be noted here that the fatty acids released from the (galacto)lipids by the lipase are not only substrate for 13-LOXs but also for 9-LOXs that catalyze the synthesis of fatty acid 9-hydroperoxides. Although this is not the topic of our review, we still briefly want to mention this activity since 9-LOX uses the same substrates as 13-LOX. The 9-hydroperoxides lead to formation of different molecules, for example nonadienal, 9-oxononanoic acid or colnelenic acid [41,42]. Furthermore, 9-hydroperoxides could be a substrate for epoxy alcohol synthase (EAS) [43]. 9-LOX derived oxylipins are reported to be induced during hypersensitive response and they accumulate after pathogen infection in tobacco and potato [44–50].

Apart from HPL, the 13-hydroperoxides are also substrates for enzymes present in other branches of the oxylipin pathway, namely, peroxygenase (POX), divinyl ether synthase (DES) and allene oxide synthase (AOS) [1,27,51–54]. Only the GLV pathway and the pathway leading via AOS to JA formation have been characterized very well. To form GLVs, 13-hydroperoxides, formed by 13-LOX, are cleaved by 13-hydroperoxide lyase(s), 13-HPL, into a C_12_ compound, 9*Z*-traumatin, and a C_6_ compound that varies, depending on the precursor: α-linolenic acid leads to the formation of *Z*-3-hexenal and linoleic acid to the formation *n*-hexanal [1,2,55,56]. Similar to LOX occurrence, beside 13-HPL there are also two other subfamilies of HPLs: 9-HPL and 9/13-HPL, which can cleave 9- or 9- and 13-hydroperoxides respectively. Both 9/13-HPLs [57–60] and 13-HPLs have been found in many different plant species [2,61–64]. The hydroperoxide lyase enzyme, 13-HPL (hereafter mentioned as HPL), was first purified from membranous fractions of tea leaves [65] and it is reported to be localized in the outer chloroplast membrane in spinach, Arabidopsis and tomato (Figure 1) [66–68]. Interestingly, natural variation also affects HPL activity. In *Arabidopsis thaliana* ecotype Columbia-0 a 10-nucleotide deletion in the coding region of the *HPL* (At4g15440) gene results in a truncated HPL without catalytic activity [69]. This deletion abolishes production of GLVs but does not seem to influence growth and development of Col-0 [70]. Other ecotypes such as Landsberg *erecta* (Le*r*), Wassilewskija (Ws), Bensheim-0 (Be-0) and Nossen-0 (No-0) have a functional copy of this *HPL* gene and do produce GLVs [69,71,72].

Finally, *Z*-3-hexenal produced by HPL is quite unstable and is converted to *E*-2-hexenal non-enzymatically or through the activity of (3Z):(2*E*)-enal isomerase [60,73]. These C_6_ aldehydes can be transformed to the corresponding alcohols and esters through the activity of alcohol dehydrogenase (ADH) and alcohol acyltransferase (AAT) [1,13] (Figure 1). A mutation in the *ADH1* gene [74,75] (At1g77120), that leads to a premature stop codon, has been used to study the role of ADH1 in GLV emission. *Adh1* mutant plants release lower amounts of hexanol and *Z*-3-hexenol compared to wt, while *E*-2-hexenal levels are clearly increased. Moreover, in *adh1* mutant plants the expression of *HPL* is higher than in wild type and this is consistent with a 50% higher HPL activity than in wild-type [72,76]. These data strongly suggest that *ADH1* is one of the enzymes converting hexanal in hexanol and *Z*-3-hexenal in *Z*-3-hexenol, respectively. The protein is predicted to be cytosolic by UniProt (http://www.uniprot.org/) and by different localization programs *in silico* (WoLFPSORT, SubLoc, LOCtree). In Arabidopsis AAT has been characterized well by *d’Auria* and co-workers as a member of BAHD acyltransferase family [77], called CoA:(*Z*)-3-hexen-1-ol acetyltransferase (CHAT) and predicted to be localized in the cytoplasm [13].

### 2.1. The Close Link between GLVs and JA: Substrate Competition or Different Regulation?

In the oxylipin pathway, HPL and AOS are the committed steps leading to the synthesis of GLVs and JA, respectively. JA and its oxylipin derivatives, named jasmonates (JAs), are key players in the regulation of induced plant responses against herbivores and necrotrophs [78]. HPL and AOS are almost identical enzymes, both belong to the CYP47 family and both rearrange hydroperoxides, albeit into structurally different products [27,64,79]. In addition, it has been shown that a single amino acid substitution changes the product specificity of AOS to HPL [80]. This high similarity of the catalytic site and the common substrate could thus lead to substrate competition.

An example of this mechanism is found in *N. attenuata*, where silencing of *HPL* activity reduced indeed the release of GLVs, but also amplified the JA burst, while silencing of *AOS* resulted in reduced JA accumulation, accompanied by an increased emission of GLVs [3]. Another example that supports substrate competition is found in Arabidopsis, where over-expression of *HPL* leads to lower levels of JA after wounding [81]. A similar scenario has also been reported for monocots. Rice plants mutated in *HPL3*, the gene responsible for GLV biosynthesis, exhibited enhanced induction of JA after herbivory [82]. Collectively, this indicates that modulation of AOS or HPL levels leads to a different metabolic flux in the other oxylipin branch.

Although AOS and HPL thus seem to compete for the same substrates, they are located in different parts in the chloroplast. HPL is targeted to the outer and AOS to the inner envelope of the chloroplast membrane in tomato and Arabidopsis [67,68,83]. In potato HPL and AOS are localized predominantly to the stroma and to the thylakoid membrane, respectively [84]. Therefore, branch specificity also arises from different sub-cellular localization of the enzymes involved. Upstream of HPL and AOS, lipases and 13-LOXs seem to be specialized in creating different cellular pools of substrates. We discussed the lipase(s) and LOXs involved in paragraph 2. Thus, these enzymes also provide a certain degree of specificity that likely leads to the formation of different pools of substrates, specific for a particular branch of the oxylipin pathway.

### 2.2. GLVs Are Emitted after Abiotic Stresses

GLVs are emitted in small quantities from unstressed plant tissue but are quickly released in large amounts after stress. Release of GLVs usually follows mechanical wounding [3,6,7] and is influenced by environmental conditions. For example, higher soil humidity influences positively *Z*-3-hexenyl acetate emission in maize. The same effect is observed by a temperature increase from 22 to 37 °C [11]. Similarly, raising the growth temperature of the monocot plant *Phragmites australis* to 45 °C results in higher emissions of *E*-2-hexenal that is maintained during and after the high-temperature treatment. In these conditions, the lipid precursors of GLVs are thus made available. At high temperatures chloroplast membranes are re-modelled according to the stress perceived [85–87], thus releasing lipids that are available to feed the GLV synthesis for longer time than the high-temperature stress episode [88]. In addition, light is an important factor that influences GLV emission. A sudden switch from light to dark determines an increased emission of C_6_ aldehydes, C_6_ alchohols and C_6_ esters in *Helianthus annuus*, *Populus alba*, *Quercus ilex* and *Dactylis glomerata* [5,9,89,90]. This induction in emission is also observed with high light intensities in e.g. *Phragmites australis* [88]. High light also causes the Arabidopsis *npq1* mutant, which is unable to carry out the xanthophyll cycle and thus more susceptible to photoinhibition, to emit *E*-2-hexenal [88]. Interestingly *npq1* treated with isoprene does not emit *E*-2-hexenal upon high light treatment. Isoprene protect cellular membranes from denaturation [91] and this indicates that during high light treatment lipids are made available for GLV biosynthesis via reorganization of membranes. Unlike the induction upon high temperature, the emission of this C_6_-aldehyde drops once the stress is removed [88]. Finally, ozone exposure has also been reported to induce the release of *Z*-3-hexenol in tobacco and maize [9].

The emission of GLVs in conditions where the leaf tissues are intact raises the question how they might be released. GLVs are formed in green organs that can exchange gaseous compounds through stomata. Although this could also be the case for GLVs, a recent paper on silver birch demonstrates that in conditions of ozone or high temperature stress stomata close and GLVs and other volatiles are still emitted [92].

### 2.3. GLVs Are Detoxified in Different Ways

The primary products of the GLV-pathway, C_6_ aldehydes are toxic to plant cells: *Z*-3-hexenal is quickly converted to the more stable *E*-2-hexenal which has an α,β-unsatured carbonyl group, highly reactive with nucleophilic atoms provided by, for example, sulphydryl (–SH) groups, common in cellular proteins [6,93]. Exogenous application of C6-aldehydes to plants in high concentrations leads to toxic effects [94] while treatment with low concentrations, without any toxic effects, leads to the conversion of their alcohols and esters [95,96] indicating that these are less toxic. This conversion also occurs upon wounding: real-time volatile analyses shows that *Z*-3-hexenal is formed immediately in wounded Arabidopsis leaves (30–45 seconds after damage), followed by the *Z*-3-hexenol and *Z*-3-hexenyl acetate (at about 5 minutes) [13]. In aspen leaves the emission of *Z*-3-hexenal is even faster within two seconds after wounding [6]. Other recent work demonstrates that the major product in completely disrupted Arabidopsis leaf tissues is *Z*-3-hexenal, while *Z*-3-hexenol and *Z*-3-hexenyl acetate are produced in the intact parts of partially wounded leaves [94]. In healthy tissue, adjacent to disrupted tissues, NAD(P)H and acetyl coenzyme A, needed to form the alcohol and acetate, respectively, are generally available [94]. *In vitro* experiments showed that *Z*-3-hexenal and its isomer, *E*-2-hexenal, have antimicrobial activity and are able to inhibit the growth of several different strains of bacteria and the pathogenic fungus *Botrytis cinerea* [71,97]. Therefore, C_6_ aldehydes might be released in disrupted tissues or at the wounding site as antimicrobial compounds to prevent microbes to invade the plant. Moreover, exogenous application of GLVs to undamaged plants results in the transcription of defense-related genes and the induction of defense-related secondary metabolites, with generally C_6_ aldehydes having stronger inducing activity [4,25,95,98–104]. Thus, production of C_6_ aldehydes at the wound site might allow the plant to switch on its defense machinery as discussed later. Conversely, *Z*-3-hexenyl acetate might act as infochemical to insects or in plant communication. Indeed, as described later, several examples of *Z*-3-hexenyl acetate-mediated plant communication have been reported [97,105,106].

Another way plants neutralize toxic compounds is to form conjugates with glutathione through the action of Glutathione *S*-tranferase (GST). Indeed, *E*-2-hexenal induces in pumpkin seedlings a subset of glutathione S-transferase (GST) genes [107]. Moreover, in Arabidopsis *E*-2-hexenal treatment as well as wounding, results in the formation of *E*-2-hexenal-glutathione adducts [96,108]. Therefore, *E*-2-hexenal is likely detoxified as well by the action of GST.

In an elegant set of experiments, using forward genetics, several Arabidopsis mutants were obtained to which *E*-2-hexenal was less toxic. For this screen, the observation was used that Arabidopsis seedlings treated with vapors of this aldehyde show a dose dependent inhibition of root growth. This inhibitory response is triggered only by *E*-2-hexenal and not by any other GLVs. The mutant *hexenal-response1*, *her1*, does not show this inhibition. The gene mutated in *her1* codes for γ-aminobutyric acid (GABA) transaminase, an enzyme of the GABA shunt responsible of the conversion of GABA into succinic semialdehyde [96]. GABA levels of *her1* mutants are thus higher than in wild-type plants. The fact that *her1* plants have high GABA levels suggests that this is involved in the responsiveness to *E*-2-hexenal possibly by helping the plants to recover faster from the stress caused by this C_6_ aldehyde. *E*-2-hexenal induces GABA accumulation in both wild-type and mutant seedlings and exogenous application of GABA increases resistance of *her1* seedlings to *E*-2-hexenal [96]. GABA is a non protein amino-acid whose accumulation has been associated with several kind of stresses [109]. Thus, GABA might be indirectly involved in the downstream mechanisms leading to detoxifying the effect of *E*-2-hexenal.

## 3. Phytohormonal Crosstalk Orchestrates Plant Defenses

The regulation of the defense network from signaling events to the activation of effective defense responses depends basically on the action of the phytohormones SA, JA and ET [78,110,111]. Since describing in detail the signaling pathways of these phytohormones is not the aim of this review, we will just summon up some of their roles in the model species Arabidopsis.

The general rule that can be inferred from studies done so far is that biotrophic pathogens are sensitive to defenses regulated by SA, necrotrops are hindered by defenses that are controlled by JA and ET [112,113], whereas herbivory triggers a response that is regulated by the JA and/or SA signaling pathway, depending on the feeding style of the herbivore [114,115].

In Arabidopsis and tomato, SA is a strong antagonist of the JA signaling pathway [116–119], and JA and ET signaling act synergistically [78,117,120]. For example, in Arabidopsis the SA pathway, induced by the biotrophic pathogen *Hyaloperonospora arabidopsidis*, suppresses JA-mediated defenses, triggered upon feeding by caterpillars of *Pieris rapae* [119]. Although the antagonistic interaction between SA and JA has been described in detail, there are also cases of synergistic actions. For example, when in Arabidopsis the SA-dependent systemic acquired resistance (SAR) pathway and JA-dependent induced systemic resistance (ISR) pathway are simultaneously activated, this results in an higher level of protection against *Pseudomonas syringae* pv. *tomato* [121]. Another example of synergistic interaction between JA and SA was found by a microarray analysis of Arabidopsis showing that some genes are induced by both treatments [122]. Furthermore, ET not only influences JA signaling but was also shown to enhance the response of Arabidopsis to SA, increasing the expression of the SA-responsive marker gene *PR-1* [123,124]. Other important factors that determine the outcome of these defense responses are the hormone concentrations and the timing of the signaling [125].

Thus, JA, SA and ET signaling pathways are deeply interconnected in a complex network of synergistic or antagonistic interactions. This network, considered the backbone of defense responses, includes also abscisic acid (ABA), auxins and gibberellins (GAs), all together influencing the final outcome of battles between pests and plants. In this already complex network, GLVs have also entered because their action influences directly JA and ET signaling pathways.

### 3.1. GLVs and JA

It has been shown that exposure to exogenous GLVs influences JA signaling in several plant species. Maize seedlings exposed to GLVs emitted by cut leaf material or by caterpillar feeding responded with a transient JA-burst 30 min later. This was also the case when seedlings were exposed to vapors of single compounds *i.e.*, *Z*-3-hexenal, *Z*-3-hexenol, *Z*-3-hexenyl acetate [105]. Exogenous application of *E*-2-hexenal or *Z*-3-hexenal to Arabidopsis plants induces the expression of *VSP1* and *AOS*, while in the JA-insensitive mutant, *jar1*, these responses are absent [102]. We have recently shown that a pre-treatment of Arabidopsis plants with *E*-2-hexenal increases susceptibility to *Pseudomonas syringae* pv. *tomato* by activating the JA-dependent signaling pathway. We also demonstrated that the presence of *HPL* positively influences JA levels and its signaling pathway, which help the bacteria to shut down SA-dependent defenses [26]. A similar influence of HPL on *Xanthomonas orizae* pv. *orizae* resistance in rice was observed recently by Tong and colleagues, who showed that the *hpl3* mutant is more resistant than the wt. *hpl3* plants show enhanced induction of JA upon herbivory and higher (three fold) basal levels of SA [82]. Thus, both AtHPL1 and OsHPL3, by affecting the levels of JA (and SA) and GLVs modulate defense responses against pathogens.

### 3.2. GLVs and Ethylene

ET is a volatile hormone crucial in mounting plant defenses. The positive interaction of ET and JA is well documented and, in Arabidopsis, these two hormones partially share downstream signaling pathways through ORA59, an AP/ERF transcription factor which integrates JA and ET signals during plant defense responses [126]. We have previously described that JA and GLVs share common substrates and they are reciprocally influenced (Sections 2.3 and 3.1). Thus, we considered the hypothesis that even ET and GLVs may influence each other. Indeed some evidence suggests an interaction: for example, Ruther and Kleier found that ethylene synergizes the emission of volatiles induced by *E*-2-hexenol [127]. Furthermore, genes involved in ethylene biosynthesis are inducible by GLVs in *P. lunatus* [128] and induction of some genes by C_6_ aldehydes is *etr1*-dependent in Arabidopsis [102]. Finally, we have recently published that *E*-2-hexenal increases susceptibility to *Pseudomonas syringae* pv. *tomato* through the activity of ORA59 [26]. Taken together these studies also indicate that GLVs likely induce some responses that are also ethylene-dependent.

## 4. GLVs and Plant Pathogens

There are several examples that GLVs are emitted by plants upon biotic stress caused by pathogens. For instance, upon infection with the pathogenic bacteria *Pseudomonas syringae* pv*. phaseolicola*, Lima bean leaves release *E*-2-hexenal and *Z*-3-hexenol [8]; *Nicotiana tabaccum* infected with *Pseudomonas syringae* pv. *syringae* emits *E*-2-hexenal [9]. In both cases the emission of these GLVs starts between 18 and 20 hours post infection, while bacteria are still growing in the exponential phase, and lasts for three days [129]. *Pseudomonas syringae* multiplies in the apoplast and it is considered a hemi-biotrophic pathogen [130]. The question rises: is the plant producing GLVs with the aim of inhibiting bacterial growth or are the bacteria inducing the plant to produce GLVs for its own benefit?

There are several data showing that GLVs have antimicrobial activity against bacteria [8,97,131,132]. This is especially true for C_6_ aldehydes, of which *E*-2-hexenal has the highest antimicrobial activity, due to the highly reactive electrophilic α,β-unsaturated carbonyl moiety [93]. Therefore, it would be favorable for the plant to produce GLVs after wounding in order to decrease the infection and inhibit the growth of bacteria [133]. The data available on antimicrobial activity of GLVs are based on *in vitro* studies. For instance, upon Pseudomonas infection, Lima bean leaves release *E*-2-hexenal and *Z*-3-hexenol in amounts sufficient to inhibit bacterial growth *in vitro* [8]. However, these *in vitro* assays do not take into account that bacteria are inside a living organism and they are not in contact just with volatiles, but have other interactions with their host and vice versa.

To fill the lack of data on the effect of GLVs on bacteria *in planta*, we studied the role of HPL, the key enzyme in GLV biosynthesis, on the disease development in a plant-bacteria interaction. We showed that HPL has a positive influence on the growth of *P. syringae* pv. *tomato* DC3000 by inducing higher JA levels in infected Arabidopsis compared to *hpl* plants [26]. Moreover a pre-treatment with *E*-2-hexenal increases the bacterial population and this effect is partially coronatine-dependent and is mediated by ORA59, a transcription factor that integrates JA and ET signaling pathways as described above [26,126]. It is well known that Pseudomonas exploits the antagonistic effect of JA on SA-dependent defenses in Arabidopsis by synthesizing coronatine, which is a structural mimic of JA-Ile, the active form of JA. [134,135]. Thus, we hypothesize that Pseudomonas benefits from inducing *HPL* by exploiting its effect on the JA pathway. Although this is a very interesting scenario, this hypothesis has to be confirmed with further investigations.

Another bacterial pathogen, *Xanthomonas orizae* pv*. orizae* which causes blight in rice, is similarly affected by an *hpl* mutation in rice [82]. In *hpl3* rice, these bacteria grow less than on wild-type rice plants with the *hpl3* mutation affecting phytohormone levels and the expression of pathogenesis related (*PR*) genes. Indeed inducible JA and constitutive SA levels are higher, as well as marker genes for these hormones, in the *hpl3* mutant. Since the resistance to bacterial blight is coordinated by the SA and JA pathways [136–138], this shows that HPL3 is involved in the defense response against this pathogen in rice.

As reported for Pseudomonas *ssp*., GLVs are also emitted after fungal infection. Several GLVs, *i.e.*, *Z*-3-hexenal, *E*-2-hexenal, *Z*-3-hexenol, *E*-2-hexenol, *Z*-3-hexenyl acetate are emitted from maize plants infected by Fusarium *spp*. starting from 3 days post-infection (dpi) and even being higher at 7 dpi [139]. *Botrytis cinerea* causes the emission of *Z*-3-hexenal, *E*-2-hexenal, and *n*-hexanal in Arabidopsis plants [71]. *Phaseolus vulgaris*, previously treated with the non-pathogenic *Pseudomonas putida*, emits more *E*-2-hexenal when infected with *Botrytis cinerea* [140].

The first work that studied the effect of GLV on fungi was published by Major *et al.*, in 1960, which reported that *E*-2-hexenal extracted from ginko leaves inhibited fungal growth [141]. In 1989 Zeringue and McCormick treated *Aspergillus flavus* with volatiles extracted from cotton leaves [142]. The treatment with some selected compounds emitted by wounded cotton leaves shows that hexanal and *E*-2-hexenal inhibited completely the growth of the fungus, while *Z*-3-hexenol did not affect the fungus. Another study in the early 90’s determined that among the volatiles produced by crushed tomato leaves, only C_6_ and C_9_ aldehydes, but not terpenoids, had an inhibitory effect on spores of *Alternaria alternata* and *Botrytis cinerea* [131]. In *Zea mays* GLVs emitted from ground kernels also inhibited growth of *Aspergillus flavus*. Moreover there is a correlation between the GLV-aldehyde content in different maize genotypes, Aspergillus resistance, and the inhibition of growth: those genotypes with reported field resistance also have a higher hexanal and total aldehyde content [143].

One study tested the impact of GLVs on *Botrytis cinerea* by analyzing the effect of the treatment of radiolabeled C_6_ aldehydes on its proteome. *E*-2-hexenal, with an α,β-unsatured carbonyl group, can modify proteins by reacting with nuclophilic groups in proteins [144]. The majority of proteins that interact with aldehydes are on the surface of the fungal tissue and in the conidial stage the incorporation rate of aldehydes is higher than in the mycelial stage. Overall C_6_ aldehydes have an influence on the fungal secretome and since the secreted proteins are important in the host interaction, C_6_ aldehydes could negatively affect the pathogenicity [145]. Interestingly, lesions formed by Botrytis are smaller on Arabidopsis seedlings treated with *E*-2-hexenal, *Z*-3-hexenal or *Z*-3-hexenol prior to fungal infection, with *E*-2-hexenal showing the highest activity [101]. Moreover, treatment with the two C_6_ aldehydes also resulted in a stronger lignification of leaf tissues [146]. This response was observed to a higher extent with *E*-2-hexenal treatment than with *Z*-3-hexenal, which correlates with the degree of resistance observed with the volatile treated seedling. Treatment with *E*-2-hexenal, *Z*-3-hexenal or *Z*-3-hexenol induces the expression of several defense related genes in Arabidopsis, among which chalcone synthase (*CHS*), caffeic acid-*O*-methyltransferase (*COMT*), glutathione-*S-*transferase 1 (*GST1*) and *LOX2* [101]. In addition, C_6_ aldehydes also induce accumulation of plant defensin 1.2 (*PDF1.2*) and *PR-3* (chitinase B) transcripts and camalexin, the characteristic phytoalexin of Arabidopsis [146,147], an important component of the Arabidopsis defenses against Botrytis [148]. *Botrytis cinerea* is a necrotrophic pathogen, which is susceptible to JA-dependent defenses, as we discussed in section 3. Some of the genes induced by *E*-2-hexenal and *Z*-3-hexenal are also induced by JA in Arabidopsis. This is for instance the case for *CHS* [149], *PDF1.2* [117,120] and *AtLOX2*, a 13-lipoxygenase, that is involved in wound-induced JA synthesis [150]. Similarly, camalexin production in Arabidopsis is JA-dependent [151]. Thus, one could speculate that, beside general defense responses, *E*-2-hexenal and *Z*-3-hexenal also induce JA-dependent responses that help Arabidopsis plants to counteract the fungus. This hypothesis is consistent with the results we found in Arabidopsis, where Pseudomonas benefits from inducing HPL by exploiting its effect on the JA pathway. To clarify the role of GLVs on the Botrytis-Arabidopsis interaction Shiojiri and colleagues used Arabidopsis *HPL* over-expressing and *HPL*-silenced lines and quantified fungal spore germination rate and hyphal length in these lines compared to No-0 wild-type plants. *HPL* over-expressing plants showed lower susceptibility to the fungal pathogen and higher emission of GLV aldehydes while *HPL*-silenced plants showed higher susceptibility and lower GLV aldehydes emission, compared to No-0 plants [10,71]. Concentrations of GLV aldehydes equivalent to those released by diseased No-0 or HPL over-expressing leaves, but not by HPL silenced leaves, inhibited both spore germination and hyphae growth *in vitro*. Moreover, with the exception of camalexin accumulation, that is higher in HPL over-expressing leaves, compared to HPL silenced or No-0 leaves, similar induction profiles were observed for other Botrytis-induced defense responses in the various plant lines [71]. Therefore, based on these observations the authors link the higher Botrytis resistance of *HPL* over-expressing to the direct antimicrobial activity of the C_6_ aldehydes rather than to their role in regulating Botrytis-induced defenses in Arabidopsis.

Overall, it seems that GLV effects on pathogenicity is different according to the kind of pathogen. Indeed GLVs help pathogenicity of bacteria such as Xanthomonas and Pseudomonas, by changing the phytohormone balance, while for the fungus Botrytis the main way of GLV action is direct toxicity. However, JA is still necessary to mount a proper defense response against *Botrytis cinerea* since the JA insensitive *coi1* mutant is more susceptible to the fungus [151].

Plants can become more resistant to pathogens when they have previously been triggered by non-pathogenic microbes in the rhizosphere. In this so-called induced systemic resistance (ISR) the plant hormones JA and ET play an important role [152,153]. Interestingly, *HPL* is one of the genes that can be triggered by such a stimulus prior to pathogen infection: Trichoderma spp. are fungi, common in soil and avirulent plant symbionts. Cucumber plants, previously infected with *Trichoderma asperellum*, show increased resistance to *Pseudomonas syringae* pv. *lachrymans* and concomitant higher *HPL* and *CHS* expression [154]. *Phaseolus vulgaris*, previously treated with *Pseudomonas putida* BTP1, emits significantly higher concentrations of *Z*-3-hexenal when infected with Botrytis. Hence, these examples confirm the link between these two phytohormones, JA and ET, and GLVs.

Finally, there is just one example, to our knowledge, where GLVs are clearly involved in virus spreading. Red raspberry, *Rubus idaeus L.*, can be infected by two viral pathogens, black raspberry necrosis virus (BRNV) and raspberry leafmottle virus (RLMV). These viruses influence the behaviour of their vector, the large raspberry aphid (*Amphorophora idaei Borner)* by inducing the plant to emit higher levels of *Z*-3-hexenyl acetate and *E*-2-hexenal. Virus-infected plants are more attractive to the aphid and the insects stay on infected plants long enough to suck the virus and spread it [155].

## 5. GLVs and Insects

### 5.1. Beneficial Insects: Indirect Defenses

Herbivore-induced plant volatiles (HIPV) can mediate indirect defenses, *i.e.*, by attracting foraging carnivorous predators and parasitoids that kill herbivores (for reviews see [156,157]). Dicke and Sabelis (1988) were the first to show that HIPVs indeed can be key foraging cues for natural enemies of herbivores. By means of a Y-tube olfactometer assay, they showed that the blind predatory mite *Phytoseiulus persimilis* depends on HIPVs for finding plants infested with its prey, the spider mite *Tetranychus urticae* [158]. Since then, many other studies have followed and they showed a similar attractive behavior of several parasitoids and predators to single volatile compounds or complex mixtures (for reviews see [156,157,159]).

As GLVs are immediately released from the wounded leaf of a plant, this group of volatiles can provide rapid and reliable information about the exact location of the attacking herbivore. However, because GLVs are released from almost every plant and under various stress conditions, they might not provide reliable information to the prey-searching carnivore. Nonetheless, many studies showed that predators and parasitoids are indeed attracted to single GLVs or to a set of GLVs [17,160–162].

Since HPL is the pivotal enzyme in the synthesis of GLVs, manipulation of its activity represents a powerful means to study the ecological function of GLVs in plant indirect defenses. In Arabidopsis, ectopic expression of *HPL* (OX-HPL) caused a significant increase in GLV production upon herbivory by white cabbage butterfly (*Pieris rapae*) larvae resulting in a higher attraction of the parasitic wasp *Cotesia glomerata*, and subsequently in a higher parasitation rate of *Pieris rapae* larvae [10]. Conversely, plants with reduced *HPL* expression released lower amounts of GLVs and attracted fewer parasitoids [10]. Arabidopsis plants which are unable to produce AOS-derived metabolites while producing increased amounts of GLVs (*aos*-OX-HPL) released hexenyl acetate as the prevalent volatile upon aphid (*Myzus persicae*) infestation [81]. In a choice assay the females of the parasitic wasp *Aphidius colemani* were significantly more attracted to the GLV-producing *aos*-OX-HPL plants, compared to *aos-hpl* plants which were unable to release hexenyl acetate upon mechanical damage [81].

Interestingly, the loss-of-function mutant *Os-hpl3* in rice was more attractive to the brown planthopper egg parasitoid, *Anagrus nilaparvatae*, albeit this plant released lower levels of GLVs. This is probably due to an increased emission of JA-dependent HIPVs since induced levels of JA were clearly increased in these plants upon herbivory [82].

In laboratory Y-tube choice assays and in field experiments *N. attenuata* plants with reduced expression of *HPL* (as*-hpl*) were less attractive to the generalist predator *Geocoris* ssp. feeding on eggs and early larval instars of the specialist lepidopteran herbivore *Manduca sexta* [163]. Another study from the same group showed that the (*Z*)/(*E*)-ratio of GLVs released from *N. attenuata* plants changed when plants were attacked by *M. sexta* caterpillars and that this herbivore-induced change in the (*Z*)/(*E*)-ratio tripled the foraging efficiency of the generalist predators *Geocoris* spp. in nature [12].

Although indirect defenses via the release of plant volatiles have been described in many cases there is until now only little evidence that an increased attractiveness of predators and parasitoids also leads to increased plant fitness. However, recently Schuman *et al.* (2012) showed that wild type *N. attenuata* plants produced twice as many buds and flowers as plants with reduced GLV emission (hemi-irLOX2) [164].

### 5.2. Non-Beneficial Insects

The volatile information that plants release into the air upon herbivory is (probably) not encrypted and can thus also be used and abused by non-beneficial insects for host plant recognition to find food, mating partners or the perfect spot to oviposit. The responses of insects to GLVs can vary tremendously, and depending on the volatile composition, the insect gender or species, insects are either attracted or repelled by certain GLV compounds or mixtures [165,166].

#### 5.2.1. GLVs Are Attractive to Insects

Field experiments with tobacco (*N. attenuata*) revealed that flea beetles (*Epitrix hirtipennis*) were more abundant on GLV-producing wild type plants compared to plants with reduced *HPL* expression (as-*hpl*) [163]. In choice experiments, three different lepidopteran species preferred wild type over GLV-deficient plants [3]. Interestingly, for the tobacco hornworm, *M. sexta*, GLVs seem to serve as feeding stimulants as they eat more and grow bigger on tobacco plants that are able to produce GLVs [3,167]. In Y-tube and greenhouse experiments the winged tea aphid (*Toxoptera aurantii*) was more attracted to plastic dummies baited with either single compounds or GLV mixtures compared to hexane baited controls [168].

GLVs are not only used to find appropriate plants to feed on, but also to find mating partners; cockchafer males (*Melolontha* sp.), swarming at dusk, use plant-derived GLVs as primary attractants for mate finding [169,170]. Similar results have been shown for the garden chafer, *Phyllopertha horticola*, as exclusively male, but not female garden chafers were attracted to (*Z*)-3-hexenol [171].

Furthermore, GLVs can have a synergistic effect on insects responding to sex pheromones. This has been shown for several coleopteran as well as lepidopteran species [172].

#### 5.2.2. GLVs Are Repellent to Insects

As the increased release of plant volatiles may indicate to the herbivore that defensive compounds in the volatile-emitting plant have been induced, or that conspecific competitors and/or natural enemies are already present [165], the release of GLVs can also have a repellent effect on herbivores. Both C_6_ aldehydes and especially their alcohols were effective in reducing tobacco aphid (*Myzus nicotianae*) fecundity [173], whereas the closely related green peach aphid *Myzus persicae*, fed on HPL-depleted potato plants (*Solanum tuberosum*), showed an almost two-fold increase in fecundity compared to wild type-fed insects [174]. However, it is not clear whether GLVs have a direct toxic or repellent effect on these aphids, or rather an indirect effect by inducing changes in the plant’s leaf chemistry. Early results from Visser and Ave [175] showed that the odor of potato plants was attractive to the Colorado potato beetle, *Leptinotarsa decemlineata*, but that individual components *E*-3-hexenol, *Z*-2-hexenol, *E*-2-hexenol or *E*-2-hexenal were not. Interestingly the addition of single GLVs to the potato odor disrupted the attraction of the potato beetle, suggesting that certain GLV ratios are important for host odor recognition.

GLVs can also have a repellent effect on insects searching for mating partners. For several bark beetles it has been shown that GLVs act antagonistically on the attraction of these insects to pheromones. The general hypothesis for this repellent behavior of bark beetles to certain GLVs is that these rather general volatiles might provide the beetles with an easy strategy to avoid several species of non-host trees simultaneously [172].

Additionally, GLVs are used by gravid moths to choose appropriate host plants for their offspring. In field experiments, undamaged control plants of *N. attenuata* received more eggs from the adult females of the tomato hornworm *M. quinquemaculata* than plants that were already damaged by conspecific caterpillars [17]. Recently, it was shown that female *Manduca* moths can even distinguish between (*Z*)- and (*E*)-isomers of GLVs. In field experiments they laid fewer eggs on *Datura wrightii* plants that were perfumed with (*E*)-isomers or low (*Z*)/(*E*)-ratios, resembling the GLV bouquet of plants that are attacked by *Manduca* caterpillars [176]. This ability to recognize changes in the GLV profile enables them to oviposit on plants that are less likely to be attacked by predators (as discussed in Section 5.1), and to avoid competing against other conspecific caterpillars for resources. Additionally, *spr2* mutants of tomato plants, which release lower amounts of unsaturated GLVs and some terpenoids, were preferred over wild type plants by ovipositing *Manduca* moths [177].

These studies reveal that GLVs play an important role for beneficial and non-beneficial insects to make informed choices. However, since GLVs are released from almost every green plant they need to be emitted in certain compositions or in specific ratios in order to provide host specific, or non-host specific information and to elicit particular responses of insects. Several studies revealed that a composition/ratio-dependent release of GLVs represents a vital component of the olfactory signal in plant-insect interactions [12,175,176,178].

## 6. GLV Perception, Downstream Signaling and “Plant Communication”

It is clear from the previous paragraphs that not only insects but also plants can perceive GLVs. Treating plants with GLVs induces the expression of several defense relates genes and downstream metabolites [25,98,99,104,128]. However, in response to GLVs plants do not always activate their defense machinery, but they can also become alerted. This particular state is defined as a “primed state” that we will discuss in the next paragraph, emphasizing especially GLV induced priming.

The mechanisms through which GLVs are perceived by plants are largely unknown. Apoaequorin, a Ca^2+^ sensitive luminescent protein [179], was used to study the early signaling events that follows the perception of volatile terpenoids and GLVs [180]. Arabidopsis expressing apoaequorin were treated with different volatiles and the fastest response was obtained with *E*-2-hexenal, which triggered an increase in cytosolic [Ca^2+^] within 3 minutes after the exposure, while *E*-2-hexenol induced a slower increase in [Ca^2+^]_c_. Moreover, pharmacological studies showed that *E*-2-hexenal causes a Ca*^2+^* influx, most likely through the production of O_2_^−^, which can activate Ca^2+^ permeable channels in the plasma membrane [181]. In tomato, exposure to GLVs, emitted upon herbivory, also results in depolarization of the plasma membrane potential (*V*_m_) [182]. *Z*-3-hexenal and *E*-2-hexenal triggered a stronger depolarization of the *V*_m_ than Z-3-hexenyl acetate [182]. The three GLVs are able to trigger already significant *V*_m_ depolarizations at 50 ppm. The same authors confirmed that *E*-2-hexenal, and also *Z*-3-hexenal and *Z*-3-hexenyl acetate, cause an increase in [Ca^2+^]_c_. Interestingly, using the *V*_m_ depolarization as read-out, they showed that the volatile blend emitted from mechanically damaged tomato leaves failed to induce a change in *V*_m_, while the blend of herbivore (*Spodoptera littoralis*)-induced volatiles did. This indicates that plants are able to discriminate between a volatile blend emitted by plants challenged by herbivores or just mechanically wounded. From this evidence, it is clear that plants perceive GLVs.

Having established that GLVs can trigger early signaling events in plants, the question rises as to which concentrations and what exposure times are necessary to trigger a response. Recently Shiojiri and colleagues published that intermittent exposure of undamaged neighboring plants, twice per week over a period of three weeks, to 140 ppt of GLVs, emitted by wounded Arabidopsis, was sufficient to trigger a response in the receiver plant [183]: when subsequently damaged, receiver plants became more attractive to the parasitic wasp *Cotesia glomerata* than damaged receiver plants that had previously been exposed to volatiles of undamaged emitter plants. Interestingly, the wasp’s preference was lost, when plants unable to produce GLVs (*hpl* introgression line) were used as receiver plants or as emitter plants thus highlighting the role of GLVs in this process. This approach is important since almost all studies showing plant responses to GLVs were done with continuous exposure and often with volatile concentrations higher than ones naturally occurring [18,103,184]. In the open field, continuous exposure is unlikely because of atmospheric instability and airflows. Shiojiri demonstrated that in Arabidopsis GLVs can be signals in plant communication even without continuous exposure and that the amount necessary to elicit a response in receiver plants is very low, in the range of 24–140 parts per trillion of volume (pptV) per each repetitive exposure. Moreover, the duration of the exposure was also tested and the intermittent exposures have to be longer than one week to elicit a defense response in receiver plants. Hence, all these evidences suggest that plant-to-plant signaling in Arabidopsis can occur under laboratory conditions. In this study, the distance between emitting and receiving plants was not tested and this is of course extremely important as it has been shown for volatile compounds in Lima beans [185], with greater distances leading to less communication.

Finally, studies in Lima bean (*Phaseolus lunatus*) showed that not only undamaged neighboring plants but also distal, yet-undamaged parts of the volatile emitter plant respond to this airborne signal. In response to herbivore damage, Lima bean releases volatile organic compounds (VOCs), among which several terpenoids and *Z*-3-hexenyl acetate, and secrets extrafloral nectar (EFN). The latter is attractive to predatory arthropods, therefore functioning in the indirect defense response [106,186]. In an elegant set of field experiments, undamaged receiver leaves were exposed to VOCs released by herbivore-challenged emitter leaves [187]. This volatile treatment both induced and primed EFN secretion in the receiver leaves, resulting in a significantly reduction in subsequent herbivore damage compared to untreated leaves [187]. An artificial blend of VOCs, resembling the natural one, was also able to induce and prime EFN secretion, and Kost and Heil (2006) subsequently identified the GLV *Z*-3-hexenyl acetate as the component in the VOC blend able to elicit this defense response [106,188]. These experiments not only demonstrate that GLVs might serve as external signals mediating within-plant communication but also that this communication occurs under natural conditions.

Therefore, in ‘plant communication’ there are two different kinds of receivers: the first one is a neighboring plant, the second one is a distal undamaged part of the emitting plant. In the latter case, the volatile signals help to induce a systemic response and this can be faster than a vascular signal molecule and can reach also organs not directly connected through vascular connections [187,189,190]. Thus, in this model vascular and airborne signals act synergistically to ensure optimal resistance in distal plant parts. Since “communication” to neighboring plants has not been proven yet to be an effective advantage for the emitter, receiving plants have been defined as eavesdroppers [18,166,191]. In a recent review, Heil and Karban analyze this aspect as well, describing in detail plant communication. They argue that in case the neighbors are offspring of the emitter plant, helping these individuals by sending an alert message, could help to increase their overall efficacy at the level of the plant population [18].

### Priming by GLVs: Staying Ready for the Battle

The definition of the verb “to prime” is to prepare something, to make it ready. For a plant “to be primed” means that it has prepared its inducible defenses in order to be able to activate them more quickly and stronger [21]. Priming can be triggered in plants by biotic stimulations, for instance by pathogenic or beneficial microorganisms, and by treatment with synthetic and natural molecules such as β-aminobutyric acid (BABA), benzothiadiazole (BTH) and SA [192–195].

BABA protects Arabidopsis against several virulent pathogens by priming resistance triggered by SA- and ABA-dependent defense mechanisms [192,196]. SA and its analogue BTH induce a particular type of priming, called systemic acquired resistance (SAR) [197]. This is a form of inducible resistance in which a first attack from a necrotizing pathogen leads to enhanced resistance to further pathogen infection in distal plant tissues [198]. The resistance conferred by SAR is effective towards a broad spectrum of pathogens. Another kind of priming-triggered resistance is ISR, which is mounted when some non-pathogenic microbes colonize the root system, triggering plant responses that reduce disease in aerial plant parts. Unlike SAR that is locally dependent on SA signaling, ISR is not associated with necrotic lesions and seems to enhance the sensitivity to JA and ET [199,200].

Plants can not only be primed directly by biotic stress, but also by environmental cues revealing that a pest or a pathogen is likely to be in their proximity. For instance, such cues can come from VOCs, emitted by infested neighboring plants or distal plant parts [106]. Additionally, it has recently been shown that also mycelia networks formed by arbuscular mycorrhizal fungi can be involved in exchanging warning signals between plants. Signals from aphid infested *Vicia faba* through mycelia networks trigger the emission of volatiles in healthy plants [201].

The first publication on volatile signaling between plants was reported by Rhoades in 1983 and describes that field-grown willows next to herbivore-attacked conspecifics are less palatable to larvae than the ones growing next to unattacked willows [202]. In the same year Baldwin and Schultz discovered that VOCs of mechanically damaged poplars and maples increased phenolics in undamaged relatives [203]. Since then, several examples of VOC-triggered priming have been reported [18].

The first study that clearly showed anti-herbivore defense priming by GLVs was conducted in maize [105]. Engelberth and colleagues demonstrated that corn seedlings previously exposed to pure GLVs or to a blend of GLVs (*Z*-3-hexenal, *Z*-3-hexenol and *Z*-3-hexenyl acetate, collected from maize plants infested by caterpillars) produce significantly more JA when mechanically wounded and treated with caterpillar oral secretions than seedlings not exposed to GLVs. Treatment with the same volatile blend failed to induce higher JA levels after only mechanical wounding, demonstrating that maize plants recognize signals coming from infested plants and use this signal for priming itself only if a biotic stress occurs.

Tobacco plants with clipped sagebrush (Artemisia) neighbors suffered less herbivory than tobacco grown in proximity of unclipped neighbors [204]. Further analysis showed that tobacco plants were primed for the levels of polyphenol oxidase (PPO), a marker of induced resistance in many solanaceous plants [191,204]. After clipping, Artemisia plants emit a few terpenoids (*i.e.*, 1,8-cineole, *E*-ocimene and *p*-cymene), a series of GLVs such as *Z*-3-hexenol, *Z*-3-hexenyl acetate, *E*-2-hexenal and methacrolein [205]. Therefore, it is difficult to state which of the emitted volatiles has a major effect on mounting this priming event. However exposure to *E*-2-hexenal or methacrolein primed *N. attenuata* plants as measured by trypsin proteinase inhibitors (TPIs) induction, upon subsequent *M. sexta* feeding, indicating a role for this GLV [205].

GLV-induced priming occurs also in woody species. In hybrid poplar *Z*-3-hexenyl acetate is the most abundant herbivore-induced GLV. Hybrid poplar leaves were exposed to *Z*-3-hexenyl acetate, at concentrations comparable to those released upon wounding or herbivory, prior to infestation with larvae of the gipsy moth *Lymantria dispar. Z*-3-hexenyl acetate primed the production of JA and linolenic acid and the expression of some oxylipin pathway genes, e.g., *LOX1*, as well as genes involved in the synthesis of anti-feeding compounds such as protease inhibitors and phytoalexins. Moreover, *Z*-3-hexenyl acetate treatment also modified the volatile emission pattern and composition, with overall higher emission of terpenoids such as *E*-β-ocimene, α-farnesene, 3[*E*]-4,8-dimethyl-1,3,7-nonatriene (DMNT) upon herbivory [23]. Terpenoids released by plants can act as semiochemicals. They enable insects to recognize host plants from a distance [206] or plants to attract predators and parasitoids of the herbivores feeding on them. Z-3-hexenyl acetate does not influence all the terpenoids emitted upon herbivory by poplar, indicating that only some terpene synthases could be primed by this GLV in poplar [23].

In a recent set of experiments Savchenko *et al.* demonstrated that both a specialist caterpillar (*Pieres rapae*) and a generalist caterpillar (*Spodoptera exigua*) actively suppress emission of GLVs in Arabidopsis, even in OX-HPL plants [207]. The relevance of this being that *P. rapae*, when given the choice, avoids GLV-primed Arabidopsis plants, thus by suppressing GLV emission the caterpillars prevent other distal parts of the plant to become primed [207].

Data available on priming by GLV volatiles are still too scarce to fully understand this complex pre-defense mechanism, especially because it is difficult to recreate natural conditions. However, it is clear that GLVs can have an important role in mounting priming responses to herbivores.

## 7. Conclusions

**Figure 2:**
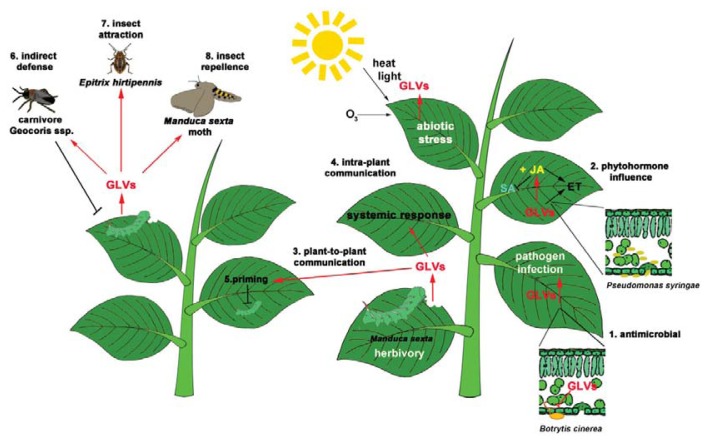
GLVs are emitted during herbivory, pathogen infection and abiotic stress. GLVs have antimicrobial activity (1), influence phytohormonal networks (2) and are involved in plant-to-plant communication (3). GLVs can trigger a systemic defense response in un-attacked leaves of the same plant (4) and induce priming in neighboring plants (5). Some carnivores e.g. Geocoris ssp., prefer plants producing GLVs, thus representing an example of indirect defense (6). GLVs can be involved in attracting herbivorous insects, e.g., *Epitrix hirtipennis* (7) or repelling herbivorous insects, e.g. *M. quinquemaculata* (8).

In this review, we highlighted that GLVs are important molecules for several aspects of plant defense systems (Figure 2). As well documented, GLVs are involved in recruiting parasitoids or carnivorous predators in order to dispose of herbivorous arthropods or to repel them. As predicted for an arms race, some herbivorous insects thus manipulate GLV emission. More recent is the finding that GLVs are also important players in plant-pathogen interactions. GLV production can influence the phytohormonal network, especially JA, ET and SA that shape the core of plant direct defense organization, and in some cases, unexpectedly, leading to higher susceptibility to pathogens in spite of the antimicrobial activity of GLVs *in vitro*. This raises the question if pathogens are capable of using GLV production to their own advantage.

In addition, GLVs can clearly prime defenses in distal parts of the plant and even in neighboring plants although this has been mostly tested in the lab. How priming is established is a wide open question since it is totally unclear how GLVs are perceived with most other aspects of GLV signaling also being unknown. It is expected that this thriving field of volatile research will try to address these issues and the role of GLVs in plant-pathogen interactions in the near future.
